# Macrophages maintain mammary stem cell activity and mammary homeostasis via TNF-α-PI3K-Cdk1/Cyclin B1 axis

**DOI:** 10.1038/s41536-023-00296-1

**Published:** 2023-05-02

**Authors:** Yu Zhou, Zi Ye, Wei Wei, Mengna Zhang, Fujing Huang, Jinpeng Li, Cheguo Cai

**Affiliations:** 1 Department of Thyroid and Breast Surgery, Zhongnan Hospital of Wuhan University; Frontier Science Center for Immunology and Metabolism, Medical Research Institute, Wuhan University, Wuhan, 430071 China; 2 Department of Thyroid and Breast Surgery, Zhongnan Hospital of Wuhan University, Wuhan, 430071 China; 3 Key Laboratory of Systems Health Science of Zhejiang Province, School of Life Science, Hangzhou Institute for Advanced Study, University of Chinese Academy of Sciences, Hangzhou, 310024 China

**Keywords:** Mammary stem cells, Stem-cell niche

## Abstract

Adult stem cell niche is a special environment composed of a variety stromal cells and signals, which cooperatively regulate tissue development and homeostasis. It is of great interest to study the role of immune cells in niche. Here, we show that mammary resident macrophages regulate mammary epithelium cell division and mammary development through TNF-α-Cdk1/Cyclin B1 axis. In vivo, depletion of macrophages reduces the number of mammary basal cells and mammary stem cells (MaSCs), while increases mammary luminal cells. In vitro, we establish a three-dimensional culture system in which mammary basal cells are co-cultured with macrophages, and interestingly, macrophage co-culture promotes the formation of branched functional mammary organoids. Moreover, TNF-α produced by macrophages activates the intracellular PI3K/Cdk1/Cyclin B1 signaling in mammary cells, thereby maintaining the activity of MaSCs and the formation of mammary organoids. Together, these findings reveal the functional significance of macrophageal niche and intracellular PI3K/Cdk1/Cyclin B1 axis for maintaining MaSC activity and mammary homeostasis.

## Introduction

Organogenesis and tissue homeostasis are tightly regulated by the cooperation of multiple cell types and signals in the microenvironment^1^. The epithelium is subjected to an array of signals present in the microenvironment, including the signals from fibroblasts^2^, immune cells^3^, endothelial cells (ECs)^4^, and other stromal cells^5^. Whether these cell types contribute to tissue development and how they orchestrate and ensure tissue homeostasis are of great interests.

The mammary gland is an epithelial organ with a highly branched tree-like pattern of ductal networks, consisting of an outer layer of elongated basal cells and an inner layer of cuboidal luminal cells^6^. Mammary stem cells (MaSCs) are located in the basal compartment^7^, and protein C receptor (Procr)^8^ and BCL11b^9^ have been identified as markers of activated and quiescent MaSCs, respectively. The mammary epithelium is surrounded by a basement membrane and is embedded in a complex microenvironment composed of multiple cell types, including immune cells, fibroblasts, nerves, vasculature, lymphatics, and adipocytes^10^. Microenvironment signals, extracellular matrix (ECM)^11^, and systemic hormones^12^ form a complex interaction network to coordinate the proper structural and functional development of the mammary gland. There is a growing appreciation of the interdependent nature of epithelial and stromal compartments during branching morphogenesis^13^. The interaction between mammary epithelial cells and stromal cells has been extensively studied, in which fibroblasts, adipocytes, immune cells and endothelial cells play essential roles through paracrine signal transduction^4,14–16^. However, due to the lack of appropriate research models in vitro, it is still hard to firmly define the crosstalk between stromal cells and mammary cells.

The mammary gland has unique postnatal development feature, most of which is achieved after birth. During puberty, hormonal signaling induces ductal elongation and branching morphogenesis^17^. In the adult, the mammary duct undergoes repeated small-scale growth and regressions with the cycling hormone levels^18^. During pregnancy, under the influences of hormones, luminal cells are induced to differentiate and develop into alveoli that produce milk at parturition^19^. Upon weaning, the mammary network involutes to a state close to that of virgin^20^. In the process of mammary development, the regulation of hormones is tightly mediated by micro-environmental cues, including ECM, stromal cells, immune cells and niche signals^21^. Therefore, it is of great interest to investigate the mediating roles of microenvironment cells and signals, which is crucial for studying mammary development.

Immune cells are widely distributed in almost all tissues, and epithelial organs in particular^22^. Epithelial tissue is the first contact point of pathogens and the first line of defense against pathogen infection. To ensure tissue homeostasis and response quickly to infection, epithelial cells collaborate closely with immune cells^23,24^. Moreover, tissue resident immune cells regulate the behavior of adult stem cells and are gradually considered to be a key component of the adult stem cell niche^25,26^. Macrophages are one of the early discovered immune cells that regulate the behavior of stem cells^27^. In particular, they are the immune cells with the highest abundance and the most extensive functions in the mammary epithelial microenvironment^28^. Macrophages appear in the mammary gland as early as the embryonic period, and continue to survive and expand throughout the lifetime^29,30^. As a professional phagocyte, resident macrophages in the mammary gland can remove dead and apoptotic cells and damaged epithelium^31^. In addition to its typical role, macrophages are also crucial for mammary development. The presence of macrophages is required for mammary duct elongation, side branch morphogenesis^32^, and alveolar formation during pregnancy^33^. Mammary “ductal macrophages” located between the basal layer and the luminal layer were recently identified as CD11c^+^ and responsive to hormones, and were found to be involved in tissue modeling after lactation and phagocytosis of alveolar cells during involution^34^. Moreover, mammary resident macrophages (M2-like macrophages) were also found to be involved in regulating the activity of MaSCs^27,35^. For example, mammary resident macrophages interact with mammary basal cells through the notch ligand Dll1 expressed by basal cells, thereby activating Notch signaling in macrophages and promoting the secretion of Wnt ligand, thus forming a feedback signal to maintain the MaSC activity^16^. Although the function of macrophages has been well studied, little is known about the intracellular molecular mechanisms induced by macrophages in mammary cells.

The capacity of three-dimensional (3D) organoid cultures to resemble a near-physiological tissue organization, which contains the correct cell types and performs the main functions of the tissue, makes organoids an excellent model for studying tissue development and disease^36^. So far, organoids have shown great application potential in regenerative medicine, gene therapy, precision medicine, and so on, opening up new avenues for these studies^37^. However, its clinical application still faces challenges, such as how to assemble organoids with their original niche, including immune cells, blood vessels, nerve systems, etc. Mammary organoids have been well established from dissociated single or re-aggregated primary mammary epithelial cells^38^. In 3D collagen gel, primary mammary epithelial cells can self-assemble into structures similar to mammary gland acini^39^. In addition, the organoids can undergo dynamic shape changes in response to growth factors and ECM cues, similar to ductal elongation and side branching seen in vivo^40^. Some organoids can maintain the expression of hormone receptors and have proved their ability to respond to hormones and drive growth, involution and milk secretion^41–46^. Organoids have provided an excellent in vitro model for studying the crosstalk between mammary epithelial cells and stromal cells in the microenvironment^47^. However, the organoid system for co-culture of mammary epithelial cells and immune cells, and for studying the crosstalk between them have not been established.

In this study, we purposely depleted macrophages in the mouse model and revealed the function of macrophages on the division and cell fate determination of mammary epithelial cells in vivo. We further established a mammary organoid system via co-culture of macrophages and mammary basal cells to facilitate the study of the regulation function of macrophages on MaSCs and mammary development. Organoids generated in this manner recapitulate mammary branching morphogenesis, and even achieve the prolactin stimulated lactation function, indicating that it is a functional external mammary gland. Mechanistically, macrophages regulate mammary development via TNF-α-PI3K-Cdk1/Cyclin B1 axis. Combining the in vivo and ex vivo approaches, we provide evidence that macrophages regulate mammary epithelial cell division and cell fate determination and thus regulate mammary epithelial pattern.

## Results

### Clodronate liposomes lead to macrophage depletion and mammary basal cell proliferation inhibition

**Fig. 1:**
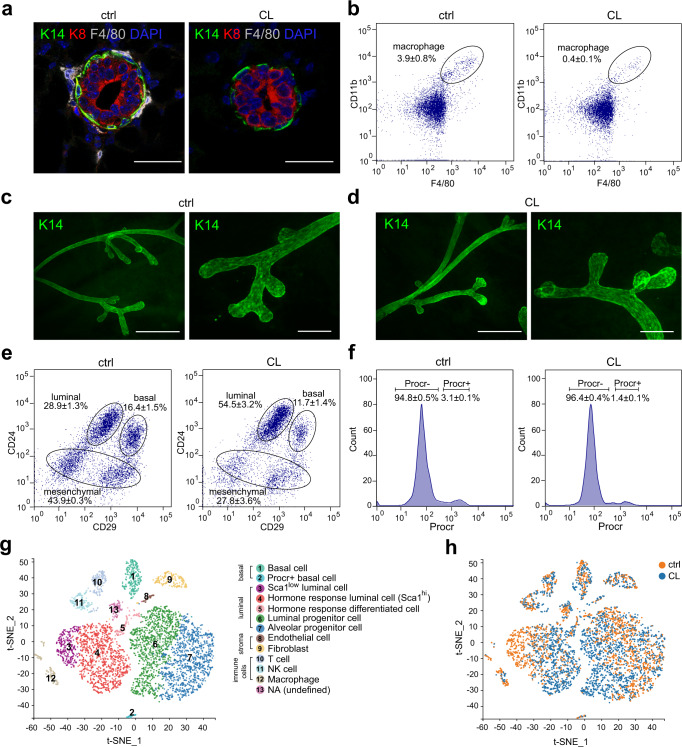
Clodronate liposomes administration induces macrophage depletion and mammary stem cell reduction. **a** Immunostaining of F4/80, K14, and K8 in mouse mammary gland. Ctrl: control mice, CL: CL treated mice, Scale bar: 20 μm. **b** Efficiency of macrophage depletion in the mammary gland measured by FACS analysis, the macrophages were labeled by CD11b^+^F4/80^+^. Data were presented as mean ± SD, and the results are representative of 3 independent experiments. Ctrl: control mice, CL: CL treated mice. **c** Whole-mount K14 staining of the mammary ducts of control mice (8 to 10-week-old), scale bars: 500 μm and 200 μm. **d** Whole-mount K14 staining of the mammary ducts of CL treated mice (8 to 10-week-old), scale bars: 500 μm and 200 μm. **e** FACS analysis for the subpopulations of the mammary epithelial cells labeled by CD24 and CD29. Data are presented as mean ± SD, and the results are representative of 3 independent experiments. **f** Procr labeled mammary stem cells (gated out of lin^−^CD24^+^CD29^hi^ basal cell population) were detected by FACS analysis in the mammary glands of control and CL treated mice. Data are presented as mean ± SD, and the results are representative of 3 independent experiments. **g** t-SNE distribution of all the mammary single cells with clusters, with colors corresponding to unbiased clustering and annotated by cell type. **h** t-SNE distribution of all the mammary single cells with samples, with colors corresponding to different samples, the cells from control mice are in blue, while the cells from CL treated mice are in orange.

To understand the functions of macrophage on mammary development and mammary epithelium cell regulation, clodronate liposomes (CL) were used to specifically remove macrophages of adult mice (8 to 10-week-old). The procedure of CL administration was illustrated in Supplementary Fig. 1a. Immunohistochemistry (IHC) and fluorescence activated cell sorting (FACS) analyses indicated that mammary resident macrophages were significantly decreased (Fig. 1a) as expected, and the depletion efficiency was about 90% (from 3.9 ± 0.8% to 0.4 ± 0.1%, *P* < 0.001) (Fig. 1b). We then investigated the mammary phenotypic changes caused by CL treatment. Whole mount staining analysis did not show any observable morphological changes of mammary epithelial ducts (Fig. 1c, d). However, FACS analysis indicated that the proportion of mammary basal cells (lin^−^CD24^+^CD29^hi^) was significantly decreased in CL treated mice compared with that in control mice (from 16.4 ± 1.5% to 11.7 ± 1.4%, *P* < 0.001), in addition, the proportion of luminal cells (lin^−^CD24^+^CD29^low^) increased to an even greater extent (from 28.9 ± 1.3% to 54.5 ± 3.2%, *P* < 0.001) and the mesenchymal cells were also significantly decreased (from 43.9 ± 0.3% to 27.8 ± 3.6%, *P* < 0.001) (Fig. 1e). Interestingly, Procr labeled multipotent mammary stem cells (MaSCs) were also significantly decreased in CL treated mice compared with that in control mice (from 3.1 ± 0.1% to 1.4 ± 0.1%, *P* < 0.001) (Fig. 1f). To further investigate the changes of mammary gland cell population under CL treatment, we performed single cell RNA sequencing, as illustrated in Supplementary Fig. 1a: CD31^+^ and Ter119^+^ cells were removed by FACS sorting, so that the remaining single cells containing epithelial cells and stroma cells were sequenced by 10× genomics. t-SNE (t-distributed stochastic neighbor embedding) analysis of 10× genomics sequencing data indicated that cells in the mammary tissue were clustered into 13 groups (Fig. 1g), the distribution of the clustered cells from the two samples were shown in Fig. 1h, and the changes of various clusters were displayed in the pie chart (Supplementary Fig. 1b, c). In line with the results of FACS and IHC, the proportion of macrophages decreased significantly (from 5.06% to 0.93%) under CL administration. In addition, there was no significant change in NK and T cells, indicating that CL has a specific macrophage depletion effect. Endothelial cells and fibroblasts, the two major stromal cell populations, were also showed no significant changes. Among mammary epithelium cells, the basal cell population (from 6.61% to 4.81%), especially the Procr-expressing MaSCs subset (from 0.93% to 0.29%) decreased significantly. Interestingly, Sca1^low^ luminal cells almost disappeared (from 15.08% to zero), while Sca1^hi^ luminal cells increased significantly (from 15.31% to 26.60%), indicating that there are much more hormone-responding luminal cells were produced under CL administration. In addition, the luminal progenitor cells increased significantly (from 19.55% to 27.86%), while the alveolar progenitor cells remained unchanged. The division and proliferation abilities of basal and luminal cells were further determined. IHC and FACS analyses indicated that Ki67^+^ basal cells in CL treated mice decreased significantly compared with that in control mice (Supplementary Fig. 1d, e, f). In contrast, Ki67^+^ luminal cells in CL treated mice increased markedly (Supplementary Fig. 1g, h, i). Together, CL administration effectively induces the depletion of mammary resident macrophages, inhibits the division of mammary basal cells, while promotes the division of luminal cells.

### Clodronate liposomes lead to attenuation of mammary stem cell activity

**Fig. 2:**
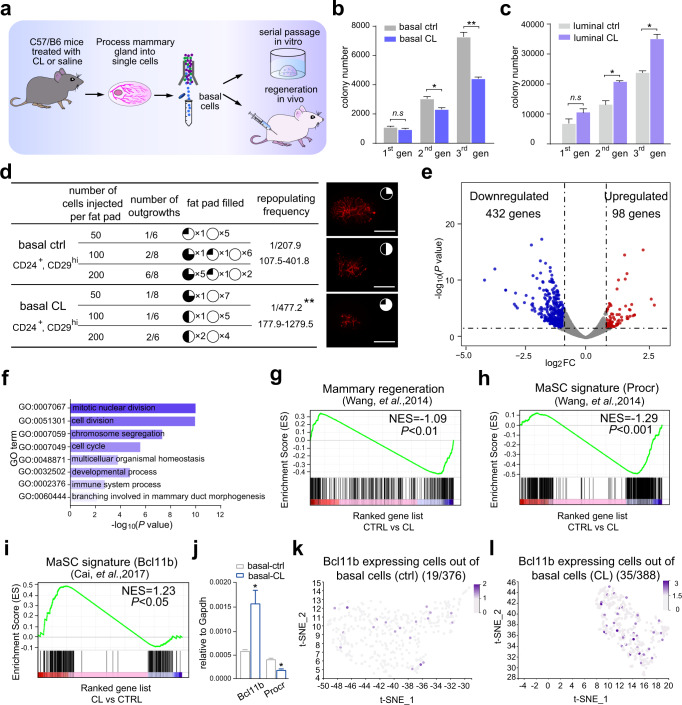
Clodronate liposomes lead to attenuation of mammary stem cell activity. **a** Schematic illustration of the serial passages in 3D culture, and the mammary regeneration assay of basal cells. **b** CL treatment inhibits basal cell proliferation: graph shows numbers of basal cell colonies in each passage. *n* = 3 replications, *P < 0.05, **P < 0.01, *n.s* represents no significance, unpaired *t*-test. **c** CL treatment promotes luminal cell proliferation: graph shows numbers of luminal cell colonies in each passage. *n* = 3 replications, *P < 0.05, *n.s* represents no significance, unpaired *t*-test. **d** Mammary gland reconstitution efficiency of basal cells in transplantation. The mammary outgrowth numbers and sizes (shown as the percentage of fat pad filled) are combined from three independent experiments. **P < 0.01, unpaired *t*-test. Scale bar: 5 mm. **e** Volcano plot of the differentially expressed genes in basal cells from control and CL treated mice. (FC > 1 and adjust *P* value < 0.05). **f** GO (Gene Ontology) analysis of the basal cells for differentially upregulated gene in CL treated mice compared with that of control mice. **g** GSEA shows that compared with the control mice, the enrichment score of the gene signature of mammary regeneration in the basal cells in CL treated mice was lower. **h** GSEA shows that compared with the control mice, the enrichment score of the gene signature of MaSC (Procr labeled) in the basal cells in CL treated mice was lower. **i** GSEA shows that compared with the control mice, the enrichment score of the gene signature of MaSC (Bcl11b labeled) in the basal cells in CL treated mice was higher. **j** qPCR analysis shows that compared with that in the control mice, the expression of *Bcl11b* in basal cells of CL treated mice was increased, *n* = 3 replications, *P < 0.05, unpaired *t*-test. **k** t-SNE plot showing the basal cells of control mice and colored by relative expression (gray = low, purple = high) of *Bcl11b*. **l** t-SNE plot showing the basal cells of CL treated mice and colored by relative expression (gray = low, purple = high) of *Bcl11b*. (Ctrl: control mice, CL: CL treated mice).

Since CL administration inhibits the division of basal cells in vivo, MaSCs are present in the basal compartment (lin^−^CD24^+^CD29^hi^), we then determined whether CL administration inhibits the activities of MaSC according to the proposed procedure (Fig. 2a). In vitro, serial colony formation analysis indicated that compared with basal cells from control mice, the number of colonies from basal cells of CL treated mice decreased significantly (Fig. 2b). In agreement with the fact that CL promotes the division of luminal cells (lin^−^CD24^+^CD29^low^), the colony number of luminal cells of CL treated mice was significantly increased in serial passages (Fig. 2c). In vivo, the regeneration efficiency of basal cells from CL treated mice was significantly lower than that from control mice (Fig. 2d). To better understand the underlying mechanism of CL regulating MaSC activity, we profiled the transcriptome of basal cells by RNA-sequencing. The volcano plot analysis showed that more than 80% differentially expressed genes (DEGs) were downregulated in CL treated basal cells (Fig. 2e). Gene ontology (GO) analysis showed that genes associated with cell cycle, cell division and developmental process were mainly included in down-regulated genes (Fig. 2f). Interestingly, gene set enrichment analysis (GSEA) revealed that gene signatures of mammary regeneration and Procr labeled MaSC were downregulated in basal cells of CL treated mice (Fig. 2g, h), while gene signature of Bcl11b labeled quiescent MaSCs^9^ were enriched after CL administration (Fig. 2i). In addition, RT-qPCR analysis confirmed that the expression of *Bcl11b* was significantly increased while expression of *Procr* was significantly decreased in basal cells of CL treated mice (Fig. 2j), suggesting that CL administration promote MaSCs from active to the resting state. In line with the result of RT-qPCR analysis, t-SNE plot analysis also showed that 19 of 376 basal cells in the control mice expressed *Bcl11b* (Fig. 2k), and 35 of 388 basal cells in the CL treatment mice expressed *Bcl11b* (Fig. 2l). Together, these results demonstrated that CL administration leads to down-regulation of stemness genes and attenuation of MaSC activities.

### Co-culture with macrophages promotes the formation of functional mammary organoids

**Fig. 3:**
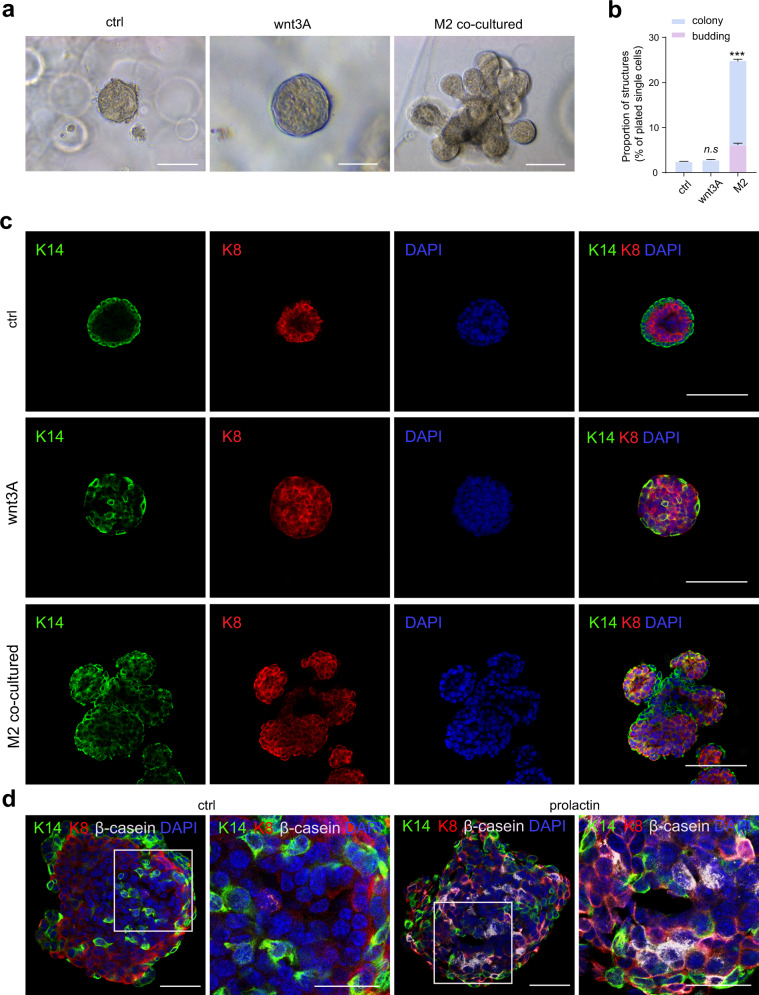
Co-culture with macrophages promotes mammary organoid formation. **a** The representative images of 3D cultured control, wnt3A treated and macrophage co-cultured basal cells. Scale bar: 100 μm. **b** Quantification of colony and budding structures derived from basal cells treated with wnt3A or co-cultured with M2. *n* = 3 replications, ***P < 0.001, two-way ANOVA. **c** Immunohistochemistry analysis indicates the representative images of colonies/organoids of cultured control, wnt3A treated and M2 co-cultured basal cells, labeled for K14 and K8. Scale bars: 50 μm. **d** β-casein staining of organoids derived from M2 co-cultured basal cells, and stimulated with or without prolactin. Scale bars: 50 μm and 20 μm.

Next, we designed an in vitro co-culture system in order to explore the interplay between macrophages and MaSCs: basal cells (lin^−^CD24^+^CD29^hi^) were isolated from the digested single cells of mouse mammary gland by FACS analysis and cultured in matrix-gel as previously reported^38^. Since mammary resident macrophages have been determined as M2-like macrophages^35^, we thus isolated bone marrow-derived macrophages (BMDMs) and induced them into M2 like subtype for co-culture analysis. The induced macrophages were seeded into the trans-well cavity and the matrix-gel with mammary basal cells was inoculated into a 24 well plate. Then the trans-well cavity was put into the well with matrix-gel. Under this condition, the macrophages were co-cultured with the basal cells in matrix-gel for 11–14 days, as illustrated in Supplementary Fig. 2a. Wnt3A can maintain the activity of MaSCs in vitro^48^, which is used here as a positive control. As expected, the colony size was significantly increased in the presence of wnt3A (Fig. 3a, Supplementary Fig. 2b). Interestingly, co-culture with M2 not only increased colony size, but also induced the formation of mammary organoids (Fig. 3b, Supplementary Fig. 2b). Statistical analysis showed that about 5.8–6.4% of the colonies sprouted buds in the presence of M2 (Fig. 3b). Keratin 14 (K14) and keratin 8 (K8) expression were examined by immunohistochemical staining (IHC) analysis, and the results showed that in the control group, K14 was expressed in the outer layer and K8 was expressed in the inner layer of colonies; In the wnt3A group, K14 was expressed in most of cells and K8 was slightly expressed in few cells of colonies; Of note, in the M2 group, K14 is expressed in the outer layer and K8 is expressed in the inner layer of organoids, indicating that these organoids recapitulate the morphology and structure of mammary ducts (Fig. 3c). It is reported that *Gata3* is expressed in luminal cells and is required during early differentiation of luminal cells^49^. RT-qPCR analysis indicated that *Gata3* expression was down-regulated in coculturing with macrophages (Supplementary Fig. 2c), suggesting that the differentiation of luminal progenitor cells was slowed down. To better understand the cell population of the colonies and organoids, we analyzed CD24 and CD29 expression by FACS analysis, and found that 87.0% of luminal cells and 6.6% of basal cells were in colonies of the control group; In wnt3A group, 86.3% of luminal cells and 7.1% of basal cells were in the colonies; In M2 group, luminal cells accounted for 81.2% and basal cells accounted for 11.8% (Supplementary Fig. 2d, e, f).

The mammary gland is a target organ of endocrine hormones, such as estrogen, progesterone and prolactin, which are essential for mammary development throughout puberty, estrous cycling and pregnancy^50^. Thus, we tested the hormone responsiveness of M2 co-cultured organoids. Sca1 labels ER^+^ luminal cells and a rare pool of bipotent basal cells in the mammary gland^51,52^, compared with control group, Sca1^hi^ luminal cells in M2 co-cultured organoids decreased significantly (from 62.1 ± 1.5% to 45.8 ± 3.1%, *P* < 0.01) (Supplementary Fig. 2g). Next, we used E2 and Pg to stimulate organoid and investigated the expression of downstream genes in Sca1^hi^ cells, as illustrated in Supplementary Fig. 2h, the expression of representative downstream genes such as *Rspo1*, *Wnt4*, *Wnt5a* and *Tnfsf11*^53,54^, in Sca1^hi^ cells of M2 co-cultured organoid were slightly higher than that in the control group (Supplementary Fig. 2i). Together, these results indicated that although the ratio of Sca1^hi^ cells in the organoid co-cultured with M2 decreased to a certain extent, it did not damage the response of Sca1^hi^ cells to E2 and Pg stimulation. We further investigated the effect of E2 and Pg on organoid morphogenesis in M2 co-culture. In the control group, under the treatment of E2 and Pg, the colony expanded significantly, while almost no budding was formed. However, under the treatment of E2 and Pg, the cells of M2 co-culture group not only expanded their colonies significantly, but also had longer branches of budding organoids (Supplementary Fig. 2j, k, l). To observe the response to prolactin, we chose to treat M2 co-cultured organoids with prolactin. Excitingly, after prolactin treatment, M2 co-cultured organoids can express β-casein, one of the main components of mammalian milk. This proves that prolactin can induce the organoid cavity of M2 group to secrete milk, but it cannot induce the colony to secrete milk (Fig. 3d), supporting that the formed organoids have the function of lactation.

Together, these results demonstrated that co-culture with M2 promotes the expansion of mammary basal cells and the generation of functional mammary organoids which retain normal hormone response capability.

### Mammary organoids exhibit robust regeneration capability

**Fig. 4:**
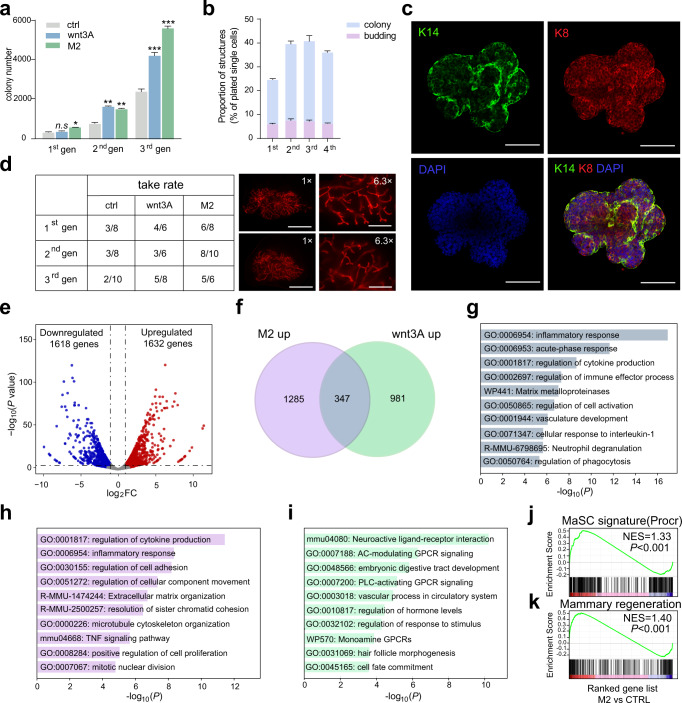
Mammary organoids exhibit robust regeneration capability. **a** Colony number of cultured basal cells in serial passages. Ctrl: control basal cell, wnt3A: Wnt3A treated basal cell, M2: basal cell co-cultured with M2. *n* = 3 replicates, *P < 0.05, **P < 0.01, ***P < 0.001, unpaired *t*-test. **b** The proportion of colonies and organoids formed in basal cells co-cultured with M2 in serial passages. *n* = 3 replicates. **c** The representative organoid images formed after 4 passages, stained for K14 and K8. Scale bar: 100 μm. **d** Organoids show a strong mammary reconstitution rate in transplantation experiments compared with that of colonies and the representative images of the reconstituted mammary glands on the right of the panel. Scale bars: 5 mm, 1 mm. **e** Volcano plot of the differentially expressed genes in organoids produced by co-culture with M2 compared with that of control (FC > 2 and adjust *P* value < 0.05). **f** Venn diagram of up-regulated overlapping genes in DEGs (differentially expressed genes) in M2 co-culture and wnt3A treated basal cells. **g** GO analysis of overlapping genes in up-regulated DEGs in M2 co-cultured and wnt3A treated basal cells. **h** GO analysis of up-regulated DEGs in M2 co-cultured basal cells. **i** GO analysis of up-regulated DEGs in wnt3A treated basal cells. **j** GSEA shows that compared with the control cells, M2 co-cultured cells were enriched with gene signatures of MaSC. **k** GSEA shows that compared with the control cells, M2 co-cultured cells were enriched with gene signatures of mammary regeneration.

Next, we asked whether the mammary organoids formed by co-culture with M2 have the ability to expand in vitro, and to regenerate mammary glands in vivo. In vitro, serial colony formation analysis showed that basal cells cultured in the presence of wnt3A or co-culture with M2 continued to expand and produce increasing numbers of colonies or organoids (Fig. 4a). Serial passage analysis also showed that the organoid morphology could be maintained with passages (Fig. 4b, c). In vivo, colonies and organoids from each passage were transplanted into cleared fat pads to assess the capability of mammary outgrowth formation. Observably, the regeneration ability of the colony in the control group decreases gradually with passages. As expected, the colonies of wnt3A treated group still maintained the regeneration ability along with passages (Fig. 4d), the images on the right panel showing the representative reconstituted mammary glands. Interestingly, organoids produced from co-cultured with M2 also maintained robust regeneration ability which better than or at least equivalent to that of wnt3A treated colonies.

To understand the changes of intracellular signals induced by co-culture with M2, we profiled the transcriptome of cultured basal cells using bulk RNA-seq. Volcanic map analysis indicated that there were 1328 significantly elevated genes induced by wnt3A, and 1632 significantly elevated genes induced by co-culturing with M2 (Fig. 4e, f). There were 347 genes up-regulated both in wnt3A treated and M2 co-cultured cells (Fig. 4f), and gene ontology analysis of the 347 genes revealed that they all induced the activation of inflammatory response and cytokine production signals (Fig. 4g). Of note, gene signatures of cell proliferation and division were enriched in organoids of M2 co-cultured cells (Fig. 4h), while was not enriched in colonies of wnt3A treated cells (Fig. 4i). GSEA indicated that gene signatures of MaSC (Procr) and mammary regeneration were enriched in M2 co-cultured cells (Fig. 4j, k). Together, these data suggested that co-culture with macrophages not only promotes the proliferation of mammary basal cells, but also maintains their regeneration capability.

### TNF-α signal activation promotes mammary organoid formation

**Fig. 5:**
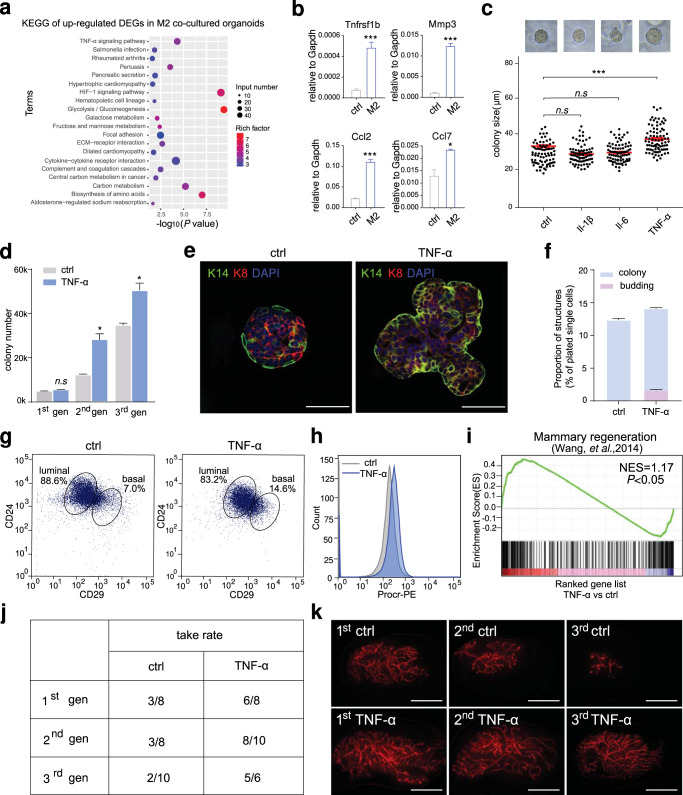
Macrophages promote organoid formation through TNF-α signaling. **a** Kyoto Encyclopedia of Genes and Genomes (KEGG) pathway analysis of the upregulated DEGs (differentially expressed genes) in M2 co-cultured cells compared with that of control cells. **b** qPCR analysis of *Tnfrsf1b, Mmp3, Ccl2, Ccl7* mRNA expression in TNF-α treated basal cells compared with that of control basal cells, *n* = 3 replicates, *P < 0.05, ***P < 0.001, unpaired *t*-test. **c** Statistics of colony size of Il-1β, Il-6 and TNF-α treated basal cells. *n* = 3 replicates, ***P < 0.001, unpaired *t*-test. **d** Changes in the number of colonies formed by cultured basal cells in the presence of TNF-α during serial passages. *n* = 3 replicates, *P < 0.05, *n.s* represents no significance, unpaired *t*-test. **e** Representative images of organoids produced by TNF-α induced basal cells. Staining with K14 and K8 antibodies. Scale bar: 50 μm. **f** Quantification of colonies and organoids formed by basal cell culture in the presence of TNF-α. *n* = 3 replicates. **g** Representative FACS analysis plots show cell populations of colonies and organoids formed by basal cell culture. The results are representative of 3 independent experiments. **h** FACS histograms shows Procr expression in organoids formed by TNF-α induced basal cells. The results are representative of 3 independent experiments. **i** GSEA shows that compared with the control cells, TNF-α treated cells were enriched with gene signature of mammary regeneration. **j** Reconstitution rate of organoids formed by TNF-α induced basal cells in serial passages. **k** Representative images of the mammary outgrowths from serial transplantation with TNF-α induced basal cells and compared with ctrl. Scale bar: 4 mm.

To understand the regulatory mechanism of macrophages on mammary basal cells, we further profiled the transcriptome of basal cells co-cultured with macrophages by RNA sequencing. KEGG analysis of up-regulated differentially expressed genes in M2 co-cultured organoids showed that the change of TNF-α signal pathway was the most significant among the top 20 enriched signal pathways (Fig. 5a). RT-qPCR analysis validated that the expression of typical downstream genes of TNF-α signal pathway, such as *Tnfrsf1b, Mmp3, Ccl2* and *Ccl7*, were up-regulated in the M2 co-cultured organoids (Fig. 5b). Next, we investigated whether TNF-α can promote the expansion of mammary basal cells and the formation of organoids. Mammary basal cells were cultured in three-dimensional matrix-gel for 7 to 11 days in the presence of TNF-α. We found that compared with the vehicle control, in the presence of TNF-α, the colony size was increased significantly (Fig. 5c), and the colony number was also increased observably in the serial passages (Fig. 5d). Interestingly, we found that with the extension of culture time to the 14^th^ day, mammary organoids were generated in the presence of TNF-α, and the ratio of colonies and organoids was similar with that of co-culture with M2 (Fig. 5e, f). FACS analysis of cultured colonies/organoids showed that the proportion of mammary basal cells was significantly increased (Fig. 5g), as well as the number of Procr^+^ basal cells (MaSCs) were also increased significantly in the presence of TNF-α (Fig. 5h). We also analyzed the effect of TNF-α on transcriptomics using RNA sequencing. GSEA revealed that TNF-α treatment is associated with the enrichment of the gene signature of mammary regeneration (Fig. 5i). Next, the results of transplantation assay further proved that the regeneration ability of the colony in the control group decreases gradually with the passage, whereas organoids formed in the presence of TNF-α maintains their regeneration capability (Fig. 5j, k). Together, these data demonstrated that TNF-α signaling activation promotes mammary organoid formation.

### TNF-α inhibition impedes the expansion of mammary basal cells

**Fig. 6:**
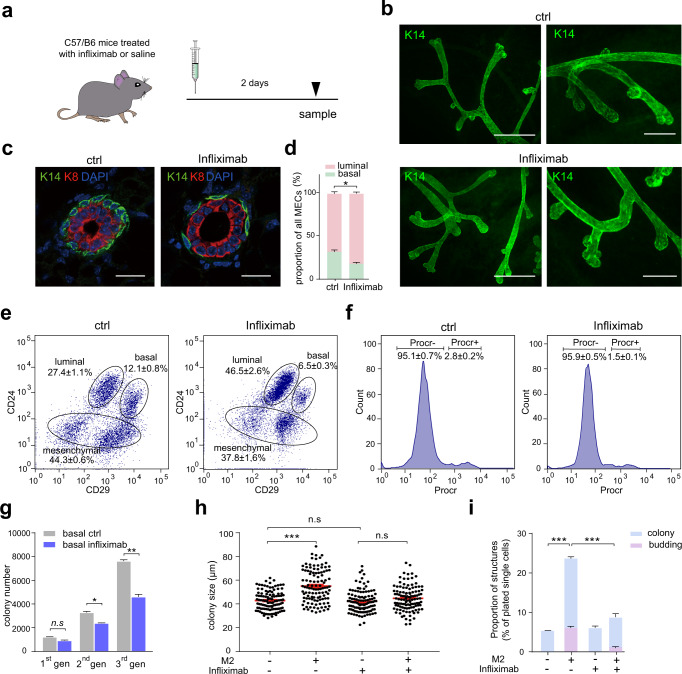
TNF-α inhibition inhibits the expansion of mammary basal cells. **a** Illustration of intraperitoneal injection of infliximab/saline in C57/B6 mice (8 to 10-week-old). **b** Whole-mount K14 staining of mammary ducts in control mice and infliximab treated mice. Scale bars: 500 μm and 200 μm. **c** Immunostaining of K14 and K8 in mammary gland (ctrl: control mice, Infliximab: infliximab treated mice). Scale bar: 20 μm. **d** Statistical analysis of the proportions of basal and luminal cells in mammary epithelial cells (MECs) in (**c**). *n* = 3 mice, *P < 0.05, unpaired *t*-test. **e** Representative FACS plots of mouse mammary epithelium cell populations (ctrl: control mice, Infliximab: infliximab treated mice). Data are presented as mean ± SD, the results are representative of 3 independent experiments, *P < 0.05, unpaired *t*-test. **f** Representative FACS plots of Procr labeled mammary stem cells (ctrl control mice, Infliximab infliximab treated mice). Data are presented as mean ± SD, the results are representative of 3 independent experiments, ***P < 0.001, unpaired *t*-test. **g** Changes in the number of colonies formed by cultured basal cells in serial passages (ctrl from control mice, Infliximab from infliximab treated mice). *P < 0.05, **P < 0.01, *n.s* represents no significance, unpaired *t*-test. **h** Colony size statistics of basal cells co-cultured with M2 and/or treated with infliximab. *n* = 3 replicates, ***P < 0.001, unpaired *t*-test. **i** Quantification of colonies and organoids formed by basal cell co-culture with BMDMs and/or treated with Infliximab. *n* = 3 replicates, ***P < 0.001, two-way AVONA.

Next, we investigated the effect of inhibiting the TNF-α signaling on mammary epithelial cells. Infliximab, a chimeric antibody that specifically binds to TNF-α and prevents its interaction with TNF-α receptors^55^, is used to inhibit TNF-α signaling in vivo and in vitro. In vivo, infliximab was administrated intraperitoneally for 2 days, as illustrated in Fig. 6a. Whole-mount staining analysis did not show any significant morphological change in the mammary epithelial ducts (Fig. 6b), while IHC analysis indicated that mammary basal cells decreased significantly under infliximab administration (Fig. 6c, d). Additionally, FACS analysis indicated that the proportions of mammary basal cells (from 12.1 ± 0.8% to 6.5 ± 0.3%, *P* < 0.001) and Procr-labeled MaSCs (from 2.8 ± 0.2% to 1.5 ± 0.1%, *P* < 0.001) were all reduced significantly, meanwhile, the proportion of mammary luminal cells increased (from 27.4 ± 1.1% to 46.5 ± 2.6%, *P* < 0.001) (Fig. 6e, f). In vitro, serial colony formation analysis indicated that the number of colonies decreased significantly in the presence of infliximab compared with that of control (Fig. 6g). We further determined whether the regulatory effect of M2 on mammary basal cells was mainly achieved through TNF-α signaling. The results showed that infliximab significantly attenuated the promotion of M2 on colony and organoid formation, as illustrated by colony size change and organoid formation (Fig. 6h, i). Together, these results demonstrated that M2 regulates MaSC activity through TNF-α signaling.

### PI3K signaling mediates the function of TNF-α on regulating MaSC activity and participates in mammary cell population shift

**Fig. 7:**
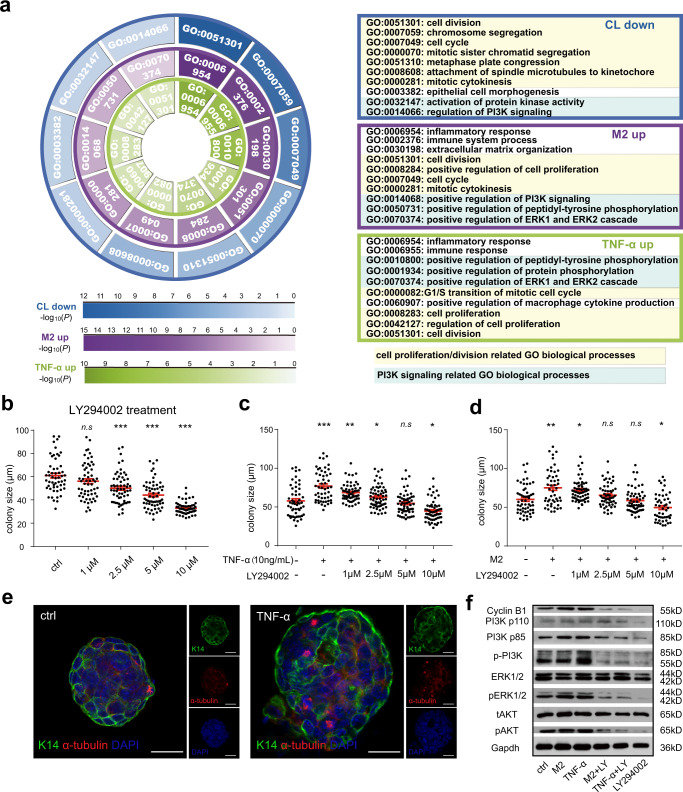
PI3K signaling mediates the function of TNF-α on regulating MaSC activity. **a** GO analysis of upregulated DEGs in organoids formed by M2 co-culture, upregulated DEGs in organoids induced by TNF-α, and downregulated DEGs in mammary basal cells from clodronate liposomes treated mice. The left part shows the representative GO terms in each group, the right part shows the definition of each GO terms. **b** Colony size statistics of basal cells treated with LY294002 at gradient concentration. *n* = 3 replicates, ***P < 0.001, *n.s* represents no significance, unpaired *t*-test. **c** Colony size statistics of basal cells treated with LY294002 at gradient concentration and TNF-α (10 ng/mL) was added for compensation. *n* = 3 replications, *P < 0.05, **P < 0.01, ***P < 0.001, *n.s* represents no significance, unpaired *t*-test. **d** Colony size statistics of basal cells treated with LY294002 at gradient concentration and M2 macrophages were co-cultured for compensation. *n* = 3 replications, *P < 0.05, **P < 0.01, *n.s* represents no significance, unpaired *t*-test. **e** Representative images of the mitosis cells in TNF-α induced basal cells compared with control. Scale bar: 20 μm. **f** Immunoblot analysis for the expression of Cyclin B1, PI3K p110, PI3K p85, p-PI3K, ERK1/2, pERK1/2, tAKT, pAKT in organoids which treated with TNF-α, co-cultured with M2, treated with TNF-α or co-cultured with M2 then followed by LY294002 treatment and treated with LY294002 separately. The results are representative of 3 independent experiments.

To identify the downstream regulators for the TNF-α signaling in regulating mammary stem cells, we profiled the transcriptome of three group cells by RNA-sequencing. First, we isolated mammary basal cells from wild type and CL treated mice, and looked for down regulated genes. Second, we collected colonies/organoids formed from control and M2 co-cultured basal cells, and looked for the up regulated genes. Third, we collected colonies/organoids formed from control and TNF-α treated basal cells, and looked for up regulated genes. Then we performed separate GO analysis of these three groups, as is shown in Fig. 7a, GO analysis of both separate data and comprehensive data of three sequencing groups indicated that biological processes related to cell proliferation/division and PI3K signaling were enriched. Accordingly, we administrated LY294002 (pan PI3K inhibitor) in basal cell colony formation assay, and the results showed that the colony size decreased in a LY294002 dose dependent manner (Fig. 7b), and LY294002 could attenuate the promotion function of TNF-α treatment and M2 co-culture (Fig. 7c, d).

Then by comparative analysis, we identified 44 genes that are low expressed in CL treated mice in vivo, and can be upregulated by TNF-α stimulation and co-culturing with M2 (Supplementary Fig. 3a). MCODE analysis suggested that Cdk1-Ccnb1 could be the key regulator of MaSC activity among the 44 genes (Supplementary Fig. 3b), which agrees with the previous reports that Cdk1-Ccnb1 plays an essential role in regulating the self-renewal of embryonic stem cells and the division of germline stem cells^56,57^. RT-qPCR were performed to validate the upregulation effect of TNF-α on the expression of Cdk1 and Ccnb1 (Supplementary Fig. 3c). Ro 3306 is a specific and selective inhibitor targeting Cdk1^58^. To examine the function of Cdk1-Cyclin B1 on MaSC activity, we cultured basal cells in the presence of Ro 3306. The results showed that the colony size is decreased in a Ro 3306 dose dependent manner (Supplementary Fig. 3d), and Ro 3306 attenuates the promotion function of TNF-α and M2 on basal colony formation (Supplementary Fig. 3e, f). Since CDK1-Cyclin B1 is a key player in cell cycle and particularly triggers mitosis^59^, we then investigated whether TNF-α could promote basal cells entering into mitosis phase. As shown the typical spindle morphology presented in Supplementary Fig. 3g, and the spindles labeled with α-tubulin in Fig. 7e, TNF-α drives more cells into mitosis compared with control (Supplementary Fig. 3h). Western blot analysis indicated that M2 co-culture and TNF-α treatment could activate PI3K signal through AKT and ERK cascades, and up-regulate Cyclin B1 expression. However, once LY294002 was administered, the phosphorylation of AKT and ERK and the expression of Cyclin B1 were all inhibited (Fig. 7e).

Next, we investigated whether PI3K signaling participated in cell population shift after CL and infliximab treatment. As illustrating in Supplementary Fig. 3i, we divided the C57/B6 mice (8 to 10-week-old, female) into three groups, three mice per each group, and treated with saline, CL and infliximab, then 1,3-Dicaffeoylquinic acid (1,3-diCQA, PI3K activator, 10 mM, 20 μL per fat pad) was injected into one of the 4^th^ mammary fat pad, and taking the other 4^th^ mammary gland as control, After 2 days of treatment, FACS analysis was performed to analyze the changes of luminal cell population. Compared with control group, 1,3-diCQA treatment leads to the significantly decrease of luminal cells (from 37.1 ± 2.4% to 30.5 ± 1.5%, *P* < 0.05) and Sca1^hi^ luminal cells (from 47.3 ± 1.1% to 34.4 ± 1.2%, *P* < 0.001), and the significantly increase of Procr^+^ basal cells (from 3.2 ± 0.2% to 5.8 ± 0.4%, *P* < 0.001), while no change of total basal cells (from 20.3 ± 0.6% to 20.9 ± 0.4%, *P* > 0.05). These results indicated that PI3K activation can inhibit the proliferation of ER^+^ luminal cells, but promote the proliferation of Procr^+^ MaSC (Supplementary Fig. 3j, k). Compared with control group, CL and infliximab treatment resulted in significant population changes in mammary epithelial cells. Macrophage depletion induced by CL treatment significantly increased the total luminal and Sca1^hi^ luminal cell populations (from 37.1 ± 2.4% to 51.6 ± 1.3%, *P* < 0.001 and from 47.3 ± 1.1% to 59.5 ± 0.3%, *P* < 0.001, respectively), and significantly reduced the basal and Procr^+^ MaSCs populations (from 20.3 ± 0.6% to 14.9 ± 0.3%, *P* < 0.05 and from 3.2 ± 0.2% to 0.9 ± 0.2%, *P* < 0.001, respectively) (Supplementary Fig. 3l). Similar to CL treatment, infliximab treatment also resulted in a significant increase in the total luminal and Sca1^hi^ luminal cell populations (from 37.1 ± 2.4% to 50.2 ± 1.1%, *P* < 0.01 and from 47.3 ± 1.1% to 65.7 ± 0.6%, *P* < 0.001, respectively), and significantly reduced the basal and Procr^+^ MaSCs populations (from 20.3 ± 0.6% to 13.3 ± 0.2%, *P* < 0.001 and from 3.2 ± 0.2% to 0.7 ± 0.1%, *P* < 0.01, respectively) (Supplementary Fig. 3m).

Next, we investigated whether PI3K activation could rescue the cell population shift induced by CL and infliximab. We found that compared with the CL treatment group, 1,3-diCQA treatment reduced luminal cells (from 51.6 ± 1.3% to 33.4 ± 0.6%, *P* < 0.001) and Sca1^hi^ luminal cells (from 59.5 ± 0.3% to 48.2 ± 1.9%, *P* < 0.001) to the level before CL treatment, and significantly increased basal cells (from 14.9 ± 0.3% to 16.6 ± 1.3%, *P* < 0.05) and Procr^+^ MaSCs (from 0.9 ± 0.2% to 2.8 ± 0.3%, *P* < 0.001) (Supplementary Fig. 3n), although they did not reach the level before CL treatment. Compared with infliximab treatment group, 1,3-diCQA treatment also reduced luminal cells (from 50.2 ± 1.1% to 32.3 ± 0.7%, *P* < 0.001) and Sca1^hi^ luminal cells (from 65.7 ± 0.6% to 49.6 ± 1.1%, *P* < 0.001) to the level before infliximab treatment, and significantly increased basal cells (from 13.3 ± 0.2% to 15.2 ± 0.4%, *P* < 0.01) and Procr^+^ MaSCs (from 0.7 ± 0.1% to 3.0 ± 0.2%, *P* < 0.001) (Supplementary Fig. 3o). Together, these results indicated that PI3K activation can rescue the mammary epithelial cell population shift induced by CL and infliximab treatment.

Taken together, these results suggested that TNF-α modulates MaSC activity and mammary epithelial cell differentiation via PI3K signaling and regulates cell division through Cdk1/Cyclin B1 axis.

## Discussion

In this study, we demonstrated the function and molecular mechanism of macrophages in promoting the proliferation of mammary basal cells and maintaining the activity of MaSCs, and established a method to obtain functional mammary organoids by co-culture with macrophages, providing a model for studying the crosstalk between mammary epithelial cells and immune cells.

Macrophages are immune cells of interest in the mammary stem cell niche. Previous studies have shown that macrophages produce Wnt ligands after Notch signaling is activated by Dll1 from MaSCs, which in return induces the MaSC activity, so they proved that macrophages are one of the important cellular components of the MaSC niche^16^. Moreover, macrophages contribute to proper ductal elongation in a manner that required STAT5 to regulate aromatase expression and estrogen production^60^. In the current study, we revealed that macrophages can maintain the activity of MaSCs and mammary homeostasis by secreting TNF-α and activating intracellular PI3K pathway in mammary cells. Therefore, we provide an intracellular signal pathway that mediates the crosstalk between macrophages and mammary cells.

TNF-α is a multifunctional cytokine, originally defined by its function of causing tumor hemorrhagic necrosis^61^, and plays a pivotal role in inflammation and autoimmune diseases^62^. TNF-α can regulate cell growth and differentiation of almost all cell types by acting alone or in concert with other cytokines, hormones or growth factors^63–65^. Previous studies have shown that TNF-α has pleiotropic functions on cellular homeostasis and stem cell activities. For example, TNF-α promotes the survival and regenerative activity of hematopoietic stem cells (HSCs) by activating a strong and specific NF-kB dependent gene program^66^. During hair follicle injury, macrophages in the wound can activate hair follicle stem cells through TNF-AKT/β-catenin signaling and promote hair follicle circulation and regeneration^67^. Interestingly, under systemic inflammation, TNF-α acts through TNFR2 and restores quiescence through TNFR1, thereby promoting the transient activation of primed neural stem cells in a cell cycle dependent manner. Therefore, TNF-α plays a dual role in driving neural stem cells into or out of quiescence by interacting with distinct receptors^68^. TNF-α also participates in regulating of several processes of mammary gland development, such as promoting mammary branch morphogenesis and alveolar differentiation and the proliferation of mammary epithelial cells^69,70^. TNF-α could promote breast cancer stem cell (BCSC) self-renewal capacity in human breast cancer cell lines through upregulating TAZ and activating non-canonical NF-κB pathway^71^. However, there are few studies on the regulation of mammary stem cells by TNF-α, and the cellular source of TNF-α in mammary gland is unclear. Our study confirms that TNF-α regulates the activity of MaSCs by activating intracellular PI3K signaling, and that mammary resident macrophages are critical source of TNF-α.

PI3K mediates the signal transduction of growth factor receptor. It can interact with RAS/RAF/MEK/MAPK pathway, and can also activate the expression of downstream AKT and mTOR genes, which makes it play a critical role in cell proliferation, metabolism, survival and cell fate determination^72^. It is reported that the activation of PI3K pathway is associated with the decreased expression and activity of estrogen receptor α (ER) in luminal B breast cancer, as well as the poor prognosis^73^. In addition, IGF1R signal maintains the self-renewal and proliferation of MaSC through PI3K/AKT/ERK pathway, suggesting the significance of PI3K signal in maintaining MaSC activity^74^. Here, we demonstrated that TNF-α induced PI3K participates in the regulation of MaSC activity, and the differentiation of ER^+^ luminal cells, providing a novel mechanism of interaction between PI3K and ER from the perspective of mammary stem cells.

Adult stem cells possess the ability to replace damaged and lost tissue cells through cell division, thus maintaining tissue homeostasis^75^. CDK1 and Cyclin B1 are important proteins involved in eukaryotic cell cycle regulation^76^. It has been reported that CDK1 is required for self-renewal of embryonic stem cells, and CDK1/Cyclin B1 complex promotes somatic reprogramming efficiency^56^. It is also been reported that CDK1/Cyclin B1 promotes cardiomyocyte proliferation and cardiac regeneration after injury^77^, as well as germline stem cell division^57^. Cdk1 expression was downregulated in aging MaSCs, and the cells exhibits attenuated proliferation and lower division frequency^78^. In addition, inhibition of Cdk1 in mammary organoids leads to decreased cell proliferation and attenuated multipotency of mammary stem cells, indicating that Cdk1 is involved in regulating the activity of MaSCs^79^. Here, we demonstrated that the Cdk1/Cyclin B1 complex in mammary epithelial cells regulates MaSCs activity, and revealed that the activation of the complex signal is dependent on TNF-α secreted by macrophages in the niche.

Mammary organoid model has emerged as a powerful tool for studying mammary development in the normal life cycle. Branch morphogenesis is a fundamental process in the mammary development^80^, while the physiological mechanism of its formation is not clear. With the help of 3D mammary organoid model, it was found that FGF2, FGF7, TGFα, FGF10 and neuregulin can promote mouse mammary branching^42,81–83^. Moreover, mammary organoid is also important tools for studying the lactation function of mammary gland in vitro. It is reported that the mammary epithelial cell line KIM2 cultured in the synthetic fat pad can form a ductal system and produce milk^84^. Interestingly, mammary organoids have been shown to undergo pregnancy-associated alveolar formation and milk production upon appropriate hormonal treatment and suffer a similar involution-like process with lactation stimulation withdraw^46^. Besides, 3D organoid models also support the study of the complex microenvironment of the mammary gland. For example, the co-culture of mammary organoids and fibroblasts helps to understand the mechanism by which fibroblasts regulate mammary branch morphogenesis through FGF-FGFR signaling^83,85,86^. In the latest study, an organoid co-culture system of endothelial cells (ECs), fibroblasts and mammary epithelial cells was established. Using this system, it was found that EC mediated Wnt signaling activation in fibroblasts is responsible for mammary epithelial patterning^4^. Here, we obtained branched functional mammary organoids by co-culturing mammary epithelial cell with macrophages, paving a new avenue for studying the crosstalk between mammary epithelial cells and immune cells.

In conclusion, our study revealed that the mammary resident macrophages play a crucial role in maintaining the division and activity of MaSCs through the TNF-α-PI3K-Cdk1/Cyclin B1 axis. Our findings provide new insights into the biological significance of macrophages in regulating stem cell activity and tissue development.

Although we have revealed a new mechanism by which macrophages regulate stem cell behavior, there is still room for improvement in our study. First, lack of genetic mouse model with TNF-α deficient macrophages, which can further firmly support our conclusion. Second, we did not further explore other factors secreted by macrophages that may participate in the regulation of mammary stem cells and their downstream signals. Third, although we have proved that PI3K signaling participated in Sca1^hi^ luminal and Procr^+^ basal cell population shift, while the current results are not enough to interpret the mechanism, more studies, such as lineage tracing and genetic operation are needed for further exploration.

## Methods

### Experiment animals

8 to 10-week-old adult wild-type female mice were used for CL (Clodronate liposomes) and infliximab administration. 3 to 4-week-old female Balb/c nude mice were used for mammary gland reconstitution assay. The mice were anesthetized in 1% Avertin in PBS (100 μL per 10 g body weight, intraperitoneal injection) before transplantation, and euthanized with 60% carbon dioxide before sampling. All animal experiments were performed according to the protocols were approved by the Animal Care and Use Committee of Medical Research Institute, Wuhan university.

### In vivo macrophage depletion

The clodronate liposomes (CL) are specifically macrophage depletion reagents and non-toxic until ingested by macrophages, once ingested, they are then broken down by liposomal phospholipases to release the drug that subsequently induces cell death in macrophages by apoptosis. For macrophage depletion in adult mice (body weight 18–20 g), we administrated Clophosome^®^ 180 μL-200 μL per mouse (F70101C-N-10, FormuMax) intraperitoneal every two days, 3 times before the mammary glands were harvested, the depletion efficiency was detected by FACS analysis.

### Immunohistochemistry

Frozen sections were hydrated with PBST: 0.1% Triton X-100 (T9284, Sigma-Aldrich) in PBS, for 5 min at first. Next, the frozen sections were blocked in blocking buffer: 10% goat serum (16210064, Thermo Fisher) + 0.1% Triton X-100 in PBS, for 2 h at room temperature. After blocking, the sections were incubated with primary antibodies at 4 °C overnight, then washed with PBST for 3 times, 5 min per time. After washing, the secondary antibodies were incubated for 1 h at room temperature, avoiding light. After secondary antibody incubation, the sections were washed with PBST 3 times, 5 min per time, finally, the sections were coated with prolong gold antifade reagent with DAPI (P36931, Invitrogen™) and covered with the cover glasses. For staining of cultured colonies/organoids, the Matrigel were de-polymerized using dispase (354235, BD Bioscience) for 15 min at 37 °C, and gently pipette up from the bottom of the well and then fixed and stained in 0.2 mL micro-centrifuge tubes. The colonies/organoids were then blocked and incubated with antibodies which refers to the frozen section staining protocol. Stained colonies/organoids were transferred to the slide, and coated with prolong gold antifade reagent with DAPI. Images were captured by laser confocal scanning microscope (ZEISS LSM880 with Airyscan).

### Mammary whole-mount staining

For whole-mount staining, the mammary glands were cut into small pieces and slightly digested (100 rpm) for 1 h in tissue digest buffer: RPMI 1640 (SH30027.01, Hyclone) + 5%FBS (P30-3302, PAN) + 1%P/S (15140-122, Thermo Fisher) + 25 mM Hepes (H4034, Sigma Aldrich) + 300 U/mL type III collagenase (LS004183, Worthington). Then, tissues were fixed in cold 4% PFA for 1 h, washed 3 times with PBST, 15 min per time, and incubated with MABT blocking buffer (with 10% FBS). 1st and 2nd antibodies were diluted in MABT blocking buffer and tissues were incubated with the antibodies for 24 h at 4 °C, after antibody incubation, washed the tissues 3 times with PBST, 15 min per time, then followed by dehydration in 80% sucrose (V900116, Sigma Aldrich) overnight before mounting. Images were captured by light microscope (ZEISS, Axios Vert. A1).

### Bone marrow-derived macrophage induction

The femur and tibia bones were collected from 6 to 8-week-old C57/B6 mice. The bones were cut open and 21 G needle and 2 mL sterilized syringes were used to flush out the bone marrow with DMEM (SH30081.LS, Hyclone) + 10%FBS + 1%P/S (3–5 mL per mouse). Then pass the collected bone marrow cells through a 70 μm strainer and centrifuge at 1000 rpm, 5 min, R.T. Remove the supernatant, add 3 mL RBC lysis (R7757, Sigma Aldrich) and incubate at R.T for 5 min to lyse the red blood cells, and add PBS to terminate the lysis process and centrifuge at 1000 rpm, 5 min, R.T. Remove the supernatant and resuspend the cells with the medium mixed with 70% DMEM (with 10% FBS + 1% P/S) + 30% L929 culture supernatant, 10 mL per mouse. For BMDM culture, the medium were changed every two days and cultured for 5–7 days. For M2-like macrophage induction, the BMDM culture medium were changed to the M2 polarization medium: complete DMEM medium + 20 ng/mL mouse Il-4 (abs04101, Absin) + 20 ng/mL mouse Il-13 (MBS1132878, MyBioSource) and cultured for 2–3 days.

### Tissue processing and mammary single cell preparation

The mammary glands were collected from 8 to 10-week-old adult mice and washed with cold PBS, and the tissues were cut into small pieces (about 1 mm^2^) and transferred into digest buffer (RPMI 1640 + 5% FBS + 1% Penicillin/Streptomycin + 25 mM HEPES), 5 mL per mouse, and digested for 2 h, 100 rpm, at 37 °C with a good shaking for every 15 min. The well-digested tissues were centrifuged at 4 °C, 1000 rpm for 5 min, and incubated with RBC lysis for red blood cell removement, then digested the cells with 0.05% trypsin (with EDTA), for 5 min at 37 °C to get single cells, and 0.1 mg/ml DNase I (D4263, Sigma Aldrich) was used to digest the free DNA for preventing cell aggregation. Then the cells were passed through a 70 μm strainer, since then the mammary single cell suspension was successfully prepared.

### FACS analysis and cell sorting

The single cell suspension was obtained from mammary glands following the protocol, for mammary epithelial cell analysis, briefly, the single cells were stained with a combination of lineage, FITC-conjugated CD45 (553080, BD Biosciences, 1:200 dilution), CD31 (553372, BD Biosciences, 1:200 dilution), TER119 (557915, BD Biosciences, 1:200 dilution), CD24-PE-Cy7 (101822, BioLegend, 1:200 dilution), and CD29-APC antibodies (102216, BioLegend, 1:200 dilution) for 20–25 min, avoid light. For Procr^+^ MaSC and Sca1^hi^ luminal cell analysis, the Procr-PE antibody (12-2012-82, eBioscience, 1:200 dilution) and Sca1-PE antibody (108107, BioLegend, 1:200 dilution) was added to the mammary epithelial cell analysis system. In the FACS sorting strategy of mammary epithelial cells, the markers used to exclude other lineages are CD45 (excluding white blood cells), CD31 (excluding vascular endothelial cells) and TER119 (excluding red blood cells). Mammary epithelial cells were labeled with CD24 and CD29. Among them, CD24 positive CD29 high (CD24^+^CD29^hi^) labeled mammary basal cells, Procr^+^ MaSCs were lin^−^CD24^+^CD29^hi^Procr^+^, while CD24 positive CD29 low (CD24^+^CD29^low^) labeled mammary luminal cells, Sca1^hi^ luminal cells were lin^−^CD24^+^CD29^low^Sca1^hi^. For Ki67 staining, after finishing the MEC staining, fixed the cells with Cytofix/Cytoperm™ (554722, BD Biosciences) for 30 min, R.T, then stained with Ki67-CF594 antibody (567719, eBioscience, 1:200 dilution) for 20–25 min. For macrophage analysis, the single cells were incubated with FcR blocking antibody (156603, BioLegend, 1:200 dilution) for 5 min at R.T, then stained with CD45-Percp Cy5.5 (550994, BD Biosciences, 1:200 dilution), CD11b-PE-Cy7 (552850, BD Biosciences, 1:200 dilution), and F4/80-APC (566787, BD Biosciences, 1:200 dilution) for 20–25 min. The living cells were gated out using DAPI staining (564907, BD Biosciences, 1:5000 dilution). All the FACS analysis were performed using Beckman cytoflex, and the statistics were processed by FlowJo_V10. The cell sorting was performed using Beckman Moflo Astrois EQ.

### Mammary gland reconstitution assay

For evaluating the basal cell reconstitution ability after macrophage depletion, the basal cells were sorted by flow cytometry, and indicated cell number was performed in each group resuspended with 50% matrix gel (354230, BD Biosciences) + 25% FBS + 25% PBS, 10 μL per fat pad, and injected into the cleared mammary fat pads of 3 to 4-week-old female Balb/c nude mice, the outgrowths were obtained and analyzed 45–60 days post-transplantation. For colony transplantation, the colonies were counted to indicated colony number before transplantation and the transplantation assay followed the protocols as described. Images were captured by a dissection microscope (LEICA, M165C).

### In vitro 3D colony formation and passage

Basal cells were sorted by flow cytometry as described. The cells were resuspended at a density of 1 × 10^6^ cells/mL in chilled matrix gel (354230, Thermo Fisher), mix well and made a drop (50 μL per 24-well-plate hole), and polymerized at 37 °C for 15 min, and then the medium was added to the plates: DMEM/F12 (Hyclone, SH30023.01) + 50 ng/mL EGF (354001, BD Biosciences) + 1:100 ITS (41440, Sigma Aldrich) for common medium and 10 ng/mL TNF-α (abs00997, Absin), 10 ng/mL Il-1β (abs04051, Absin), 10 ng/mL Il-6 (abs04084, Absin), 1 μM E2 (50-28-2, MedChemExpress), 2.5 μM Pg (57-83-0, MedChemExpress), 5 μg/mL prolactin (50367-M08B, Sino Biological), 10 ng/mL infliximab (170277-31-3, MedChemExpress) were added when needed, medium change every two days, and it usually takes 7–14 days until the colonies were harvested for measurement. For colony passage, the matrix gel was first depolymerized using dispase, then the colonies were digested into single cells using 0.25% trypsin (25200056, Thermo Fisher), and then the single cells were replayed in the matrix-gel, as described previously.

### Co-culture assay

The basal cells were cultured in 3D as described. For the M2 co-culture assay, the M2-like macrophages were digested with 0.25% Trypsin and count, then resuspended with a 3D culture medium at a density of 1 × 10^5^ cells/mL, add 200 μL BMDM suspension into each transwell chamber (0.4 μm, for 24 well-plates) and insert the chambers carefully into the 24 well-plates, the co-culture assay usually takes 11–14 days for the organoid formation. The macrophages were changed every two days.

### RNA isolation, RNA sequencing and quantitative real-time PCR

Total RNA was isolated from FACS sorted basal cells and cultured colonies/organoids lysed with RNAiso Plus (9109, Takara) according to the manufacturer’s instructions. Total mRNA concentration was determined with Nanodrop2000 and RNA-seq libraries were prepared according to the manufacturer’s instruction followed by sequencing on Hiseq X 10 sequencer (Illumina), which was performed by Wuhan Seqhealth Co., Ltd. Expression of selected genes was validated by qPCR. The cDNA was generated from equal amounts of RNA using the PrimeScript RT Master Mix (RR036A, Takara). qPCR was carried out on a FastStart Universal SYBR Green Master Mix (04913850001, Roche) on a CFX Connect Real-Time PCR Detection System (Bio-Rad). RNA level was normalized to *Gapdh*. All primer pairs were designed for the same cycling conditions: 10 min at 95 °C for initial denaturing, 40 cycles of 15 s at 95 °C for denaturing, 1 min at 60 °C for annealing and extension, following by melt curve test. Primers for qPCR are listed in Supplementary Table 1.

### Protein extraction and western blotting

Primary mammary epithelial cells were treated with lysis buffer: RIPA (P0013B, Beyotime) + 1 mM PMSF (ST505, Beyotime) for 30 min on ice, then centrifuge at 4 °C for 15 min, 12,000 rpm, the supernatant was collected into clean 1.5 mL centrifuge tubes, then boiled in a water bath for 10 min for protein denaturation. Western blot analysis was performed using the standard protocol. All blots derive from the same experiment and that they were processed in parallel.

### Single-cell RNA sequencing

FACS stored cells were resuscitated rapidly and examined by microscope after 0.4% Trypan blue coloring. When the viability of cells was higher than 80%, the experiment of library construction was performed. Briefly, single cells, reagents, and Gel Beads containing barcoded oligonucleotides were encapsulated into nanoliter-sized GEMs (Gel beads in the emulsion) using the GemCode Technology. Lysis and barcoded reverse transcription of polyadenylated mRNA from single cells were performed inside each GEM. Post RT-GEMs were cleaned up and cDNA was amplified. cDNA was fragmented and fragments end were repaired, as well A-tailing was added to the 3’ end. The adaptors were ligated to fragments which were double sided SPRI selected. Another double sided SPRI selecting was carried out after sample index PCR. The final library was quality and quantitated in two methods: check the distribution of the fragments size using the Agilent 2100 bioanalyzer, and quantify the library using real-time quantitative PCR (QPCR) (TaqMan Probe). The final products were sequenced using the DNBSEQ^TM^ platform.

### Bioinformatics analysis

GSEA v4.1.0 was used to perform the GSEA on various functional and/or characteristic gene signature. Metascape online tool (http://metascape.org) was used to perform the GO (Gene Ontology), KEGG (Kyoto Encyclopedia of Genes and Genomes) and PPI (Protein-Protein Interaction) analysis. The repopulating frequencies for mammary gland reconstitution assay were calculated using the “statmod” software package for the R computing environment (http://www.r-project.org/). For single cell RNA-seq analysis, Dr.Tom network platform of BGI was used (http://report.bgi.com).

### Statistical analysis

Data analyses were performed using GraphPad Prism 8 software. Data were presented as mean ± SD or mean ± SEM unless specified otherwise. *P* values were obtained by unpaired Student’s *t*-test or two-way ANOVA, and *P* ≤ 0.05 was considered statistically significant. The in vitro experiments were repeated independently three times with consistent results.

## Supplementary information

Supplementary information

## Data Availability

The datasets of RNA-seq for CL treated basal cells versus ctrl basal cells analysis generated during this study is available at GEO: GSE210268, datasets of RNA-seq for M2 co-cultured basal colonies versus ctrl basal colonies is available at GEO: GSE210379, datasets of RNA-seq for TNF-α treated basal colonies versus ctrl basal colonies is available at GEO: GSE210643, the datasets of single RNA-seq for CL treated mammary cells versus ctrl is available at GEO: GSE210825. All other data supporting the findings of this study are available from the corresponding author on request.

## Code availability

Only public software and custom code were used to generate or process datasets in this work. The software version and websites were described in the “Methods” section.
