# Supplementary material for: Macrophages maintain mammary stem cell activity and mammary homeostasis via TNF-α-PI3K-Cdk1/Cyclin B1 axis
Source: NPJ Regen Med. 2023 May 2;8:23. doi: 10.1038/s41536-023-00296-1 (PMC10154328; doi:10.1038/s41536-023-00296-1)
Supplement: Supplementary file 1 — Supplementary information [file 41536_2023_296_MOESM1_ESM.pdf]

Supplementary Information

Supplementary Figures and Legends

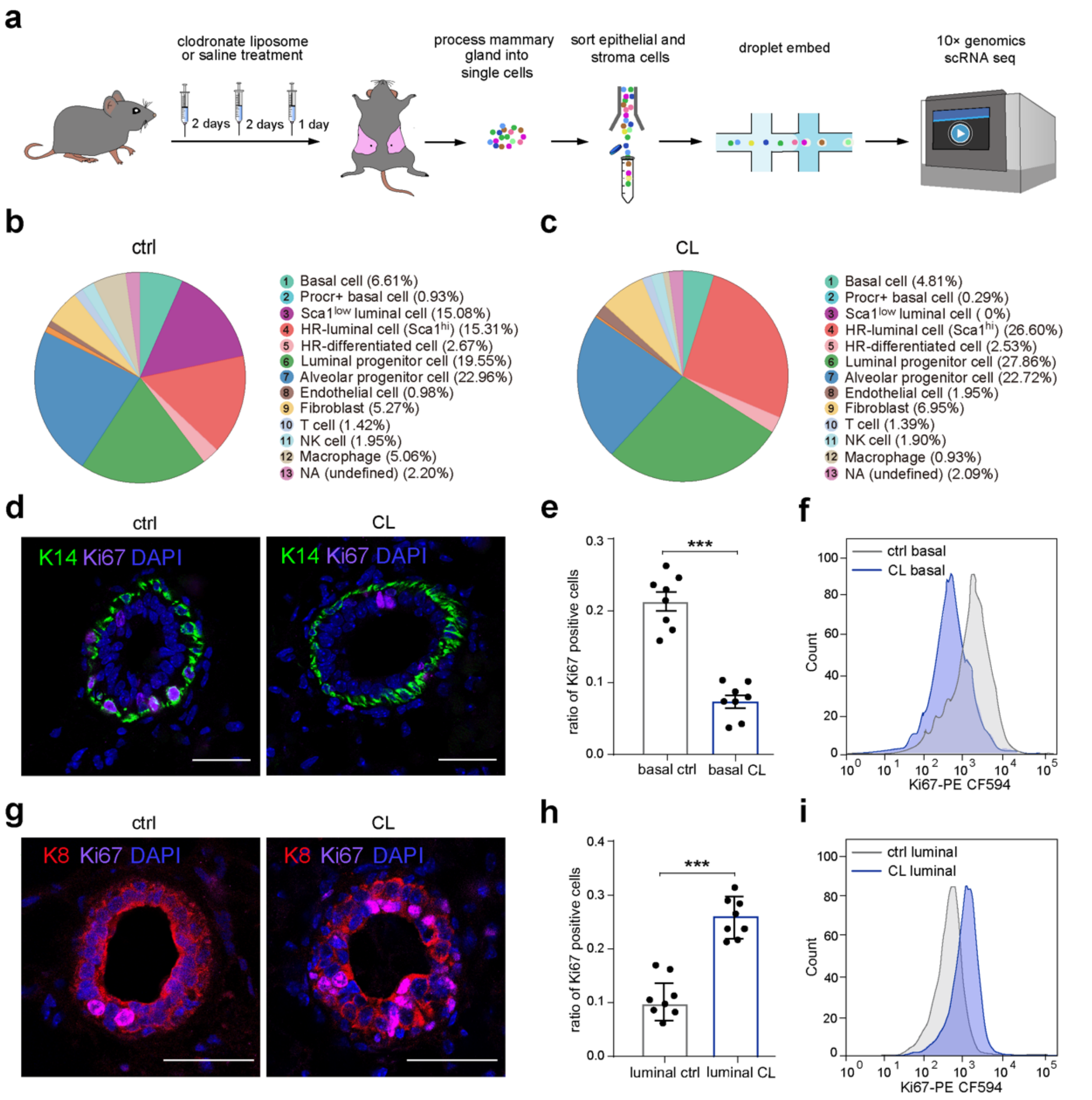

Supplementary Figure 1. Clodronate liposomes (CL) administration inhibits the proliferation of basal cells while promotes the proliferation of luminal cells. a Schematic illustration of intraperitoneal injection of clodronate liposomes and single-cell RNA sequencing. **b** The pie chart shows the proportion of various cell clusters of the mammary glands from control mice. **c** The pie chart shows the proportion of various cell clusters of the mammary glands from CL treated mice. **d** Immunostaining of Ki67 and K14 in mouse mammary gland. Ctrl: control mice; CL: CL treated mice, Scale bar: 20μm. **e**

Statistics of the proportion of Ki67 positive cells in basal cells in (**d**), n = 3 mice and 8 images. Data are presented as mean ± SEM. ***$P < 0.001$, unpaired t-test. (**f**) FACS analysis of Ki67 expression in basal cells. The results are representative of 3 independent experiments. **g** Immunostaining of Ki67 and K8 in mouse mammary gland. Ctrl: control mice; CL: CL treated mice, Scale bar: 50μm. **h** Statistics of the proportion of Ki67 positive cells in luminal cells in (**g**). n = 3 mice and 8 images. Data are presented as mean ± SEM. ***$P < 0.001$, unpaired t-test. **i** FACS analysis of Ki67 expression in luminal cells. The results are representative of 3 independent experiments.

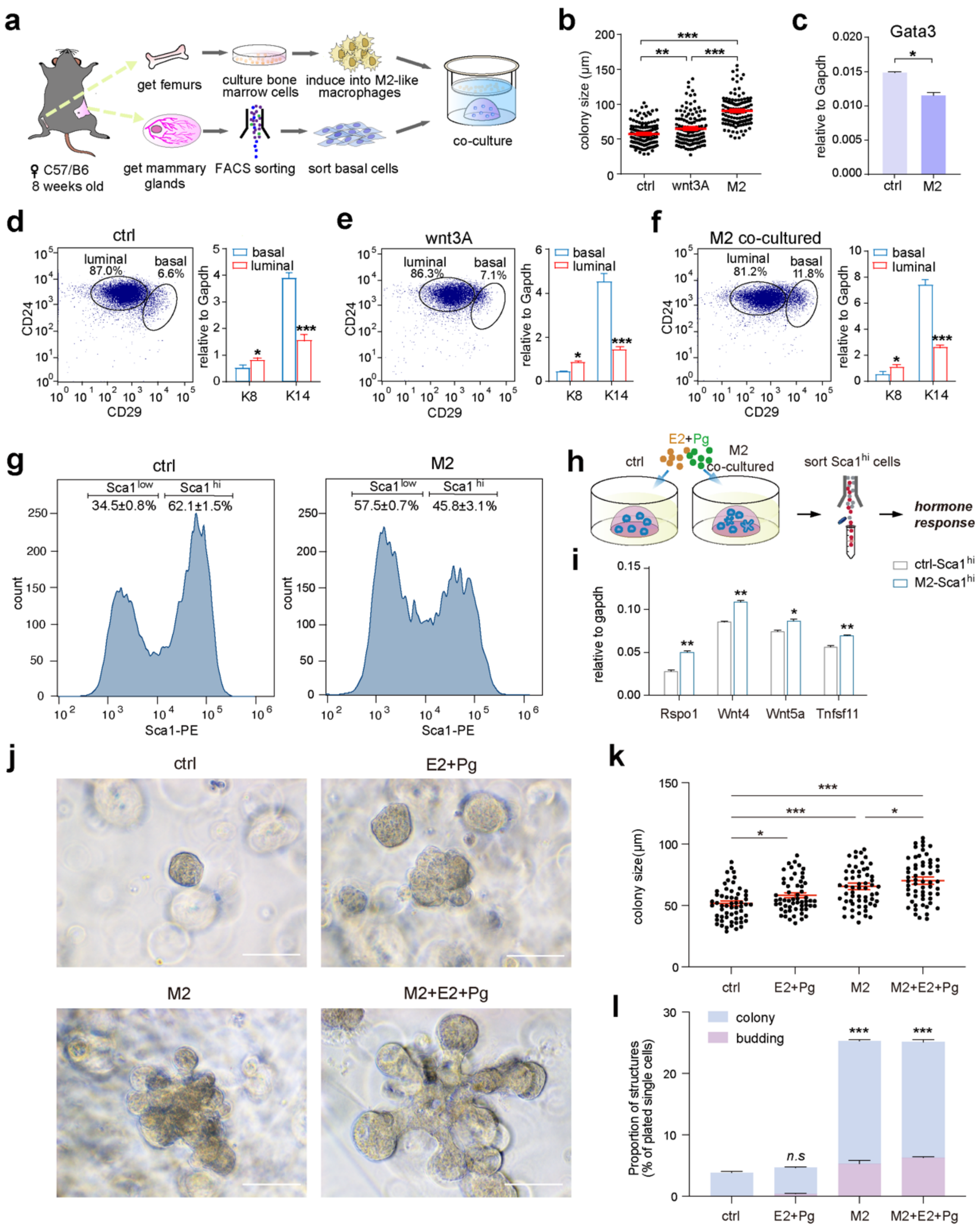

Supplementary Figure 2. Cell population analysis and hormone response detection of cultured basal cells. a Schematic illustration of the co-culture system of basal cells with bone marrow-derived macrophages (M2). **b** Quantification of the colony size of the basal derived colonies treated with Wnt3A or co-cultured with M2, compared with that of control. n = 3 replications, $**P < 0.01$, $***P < 0.001$, unpaired t-test. **c** qPCR analysis of *Gata3* expression

in M2 co-cultured organoids compared with that of control, n = 3 replications, *$P < 0.05$, unpaired t-test. **d-f** FACS analysis shows cell populations of digested colonies and organoids. qPCR analysis shows K14 and K5 expression of the colonies and organoids. Control basal cells (**d**); Wnt3A treated basal cells (**e**); M2 co-cultured basal cells (**f**). The results are representative of 3 independent experiments. n = 3 replications, *$P < 0.05$, ***$P < 0.001$, unpaired t-test. **g** FACS analysis shows $Sca1^-$ and $Sca1^+$ cell proportion in control colonies and M2 co-cultured organoids. Data are presented as mean ± SD, the results are representative of 3 independent experiments. **h** Schematic illustration of E2 and Pg treatment with control colonies and M2 co-cultured organoids for hormone response detection. **i** qPCR analysis of *Rspo1*, *Wnt4*, *Wnt5a* and *Tnfsf11* in sorted $Sca1^+$ cells from control colonies and M2 co-cultured organoids. n=3 replications, *$P < 0.05$, **$P < 0.01$, unpaired t-test. **j** Representative images of 3D cultured control, E2 and Pg treated, M2 co-cultured and treated with E2 and Pg after M2 co-cultured basal cells, Scale bars: 100μm. **k** Quantification of the colony size of the basal derived colonies treated with E2 and Pg, co-cultured with M2, or treated with E2 and Pg after co-cultured with M2, compared with that of control. n = 3 replications, *$P < 0.05$, ***$P < 0.001$, unpaired t-test. **l** Quantification of colony and budding structures derived from basal cells treated with E2 and Pg, co-cultured with M2, or treated with E2 and Pg after co-cultured with M2, compared with that of control. n = 3 replications, ***$P < 0.001$, two-way ANOVA.

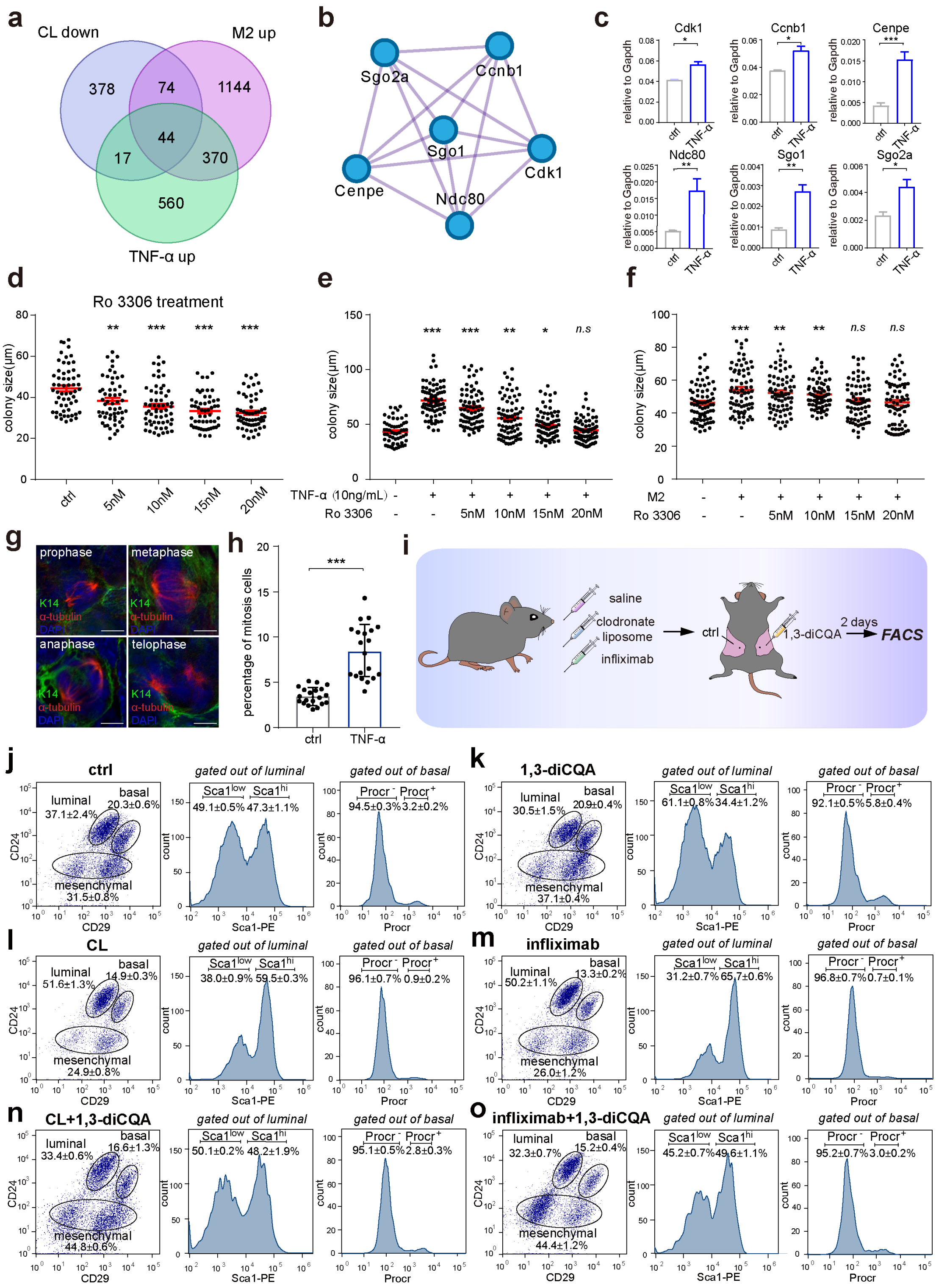

Supplementary Figure 3. Cdk1/Cyclin B1 involved in cell division regulation in MaSCs and PI3K signaling participated in cell population shift. a Venn diagram displays the overlapping genes of upregulated DEGs in organoids formed by M2 co-culture, upregulated DEGs in organoids induced

by TNF-α, and downregulated DEGs in mammary basal cells from clodronate liposomes treated mice. **b** MCODE out of Protein-Protein-Interaction (PPI) showing the certain functional module of the overlapping genes in (**a**). **c** qRT-PCR analysis for the expression of *Cdk1, Ccnb1, Cenpe, Ndc80, Sgo1 and Sgo2a* genes in organoids induced by TNF-α. n = 3 replicates, * $P < 0.05$, ** $P < 0.01$, ***$P < 0.001$, *n.s* represents no significance, unpaired t-test. **d** Colony size statistics of basal cells treated with Ro 3306 at gradient concentration. n = 3 replicates, **$P < 0.01$, ***$P < 0.001$, unpaired t-test. **e** Colony size statistics of basal cells treated with Ro 3306 at gradient concentration and TNF-α (10ng/mL) was added for compensation. n = 3 replications, *$P < 0.05$, **$P < 0.01$, ***$P < 0.001$, *n.s* represents no significance, unpaired t-test. **f** Colony size statistics of basal cells treated with Ro 3306 at gradient concentration and M2 macrophages were co-cultured for compensation. n = 3 replications, *$P < 0.05$, **$P < 0.01$, ***$P < 0.001$, *n.s* represents no significance, unpaired t-test. **g** Representative images of the spindles (marked by α-tubulin) and nucleus (marked by DAPI) in different stages of mitosis in basal colonies, Scale bar: 5μm. **h** Statistics of the percentage of mitosis cells in TNF-α induced basal cells compared with control. n = 20 colonies, ***$P < 0.001$, unpaired t-test. **i** Schematic illustration of 1,3-diCQA intra fat pad administration after saline/clodronate liposomes/infliximab treatment. **j-o** FACS analysis show mammary epithelial cell population labeled by CD24 and CD29, Sca1 labeled luminal cells, Procr labeled basal cells under separate and dual treatment with 1,3-diCQA, clodronate liposomes, infliximab. Control, treated with saline (**j**); 1,3-diCQA treatment (**k**); clodronate liposomes treatment(**l**); infliximab treatment (**m**); 1,3-diCQA intra fat pad administration

after clodronate liposome treatment (**n**); 1,3-diCQA intra fat pad administration after infliximab treatment (**o**).

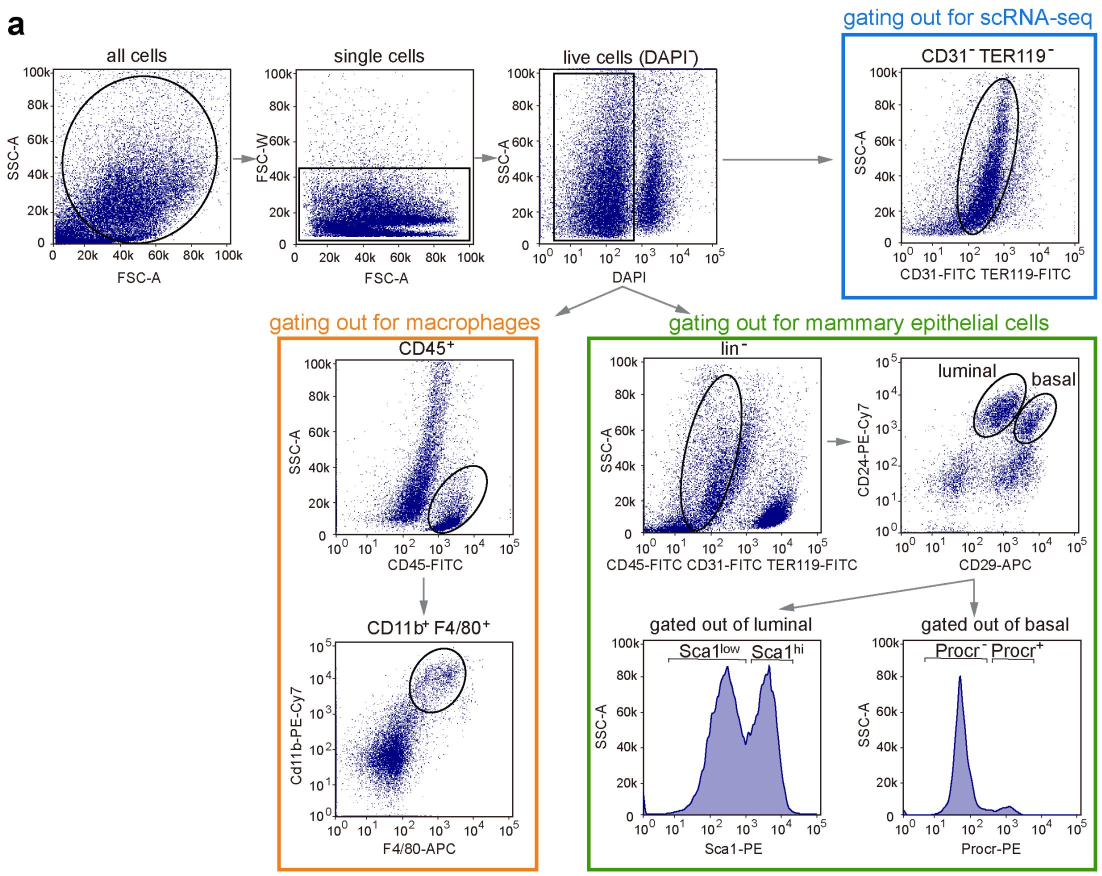

Supplementary Figure 4. FACS sequential gating strategies. a The cells sorted for scRNA-seq were $CD31^{-}TER119^{-}$ gated out of live cells correspond to supplementary Fig.1a, the gating strategy for macrophages was $CD11b^{+}F4/80^{+}$ gated out of $CD45^{+}$ cells correspond to Figure 1b, the gating strategies for mammary epithelial cells were as follows: luminal cells ($CD24^{+}CD29^{low}$) and basal cells ($CD24^{+}CD29^{hi}$) were gated out of lin^{-} cells correspond to Figure 1e and Figure 6e; according to the expression of Sca1, the luminal cells could be divided into $Sca1^{low}$ and $Sca1^{hi}$ correspond to supplementary Fig.3j-o, according to the expression of Procr, the basal cells could be divided into $Procr^{-}$ and $Procr^{+}$ correspond to Figure 1f, Figure 6f and supplementary Fig.3j-o.

Supplementary Tables

Supplementary Table 1. Primers for qRT-PCR

Bcl11b	F: CCCGACCCTGATCTACTCAC	*Cenpe*	F: GAGCACCAAGAAACTATTGACCG
	R: GGAGGTGGACTGCTCTTGT		R: CACGTTGACGCTCACTTGTCT
Gata3	F: CTCGGCCATTCGTACATGGAA	*Ndc80*	F: GCCTCTCTATGCAGGAGTTAAGG
	R: GGATACCTCTGCACCGTAGC		R: CGGTTTGTGTGTACTCAGCTT
K14	F: AGCGGCAAGAGTGAGATTTCT	*Sgo1*	F: ACGCGTAACGCAGAATTTCG
	R: CCTCCAGGTTATTCTCCAGGG		R: AATTCATCTCGGCCACTCAGG
K8	F: TCCATCAGGGTGACTCAGAAA	*Sgo2a*	F: ACCTGAATCCTGGTCTTCGC
	R: CCAGCTTCAAGGGGCTCAA		R: AGGAGTGGCTACCGAGAAGG
Tnfrsf1b	F: ACACCCTACAAACCGGAACC	*Rspo1*	F: GGGATCAAGGGCAAGAGACAG
	R: AGCCTTCCTGTCATAGTATTCCT		R: CTGGCGGATGTCGTTCCTC
Mmp3	F: ACATGGAGACTTTGTCCCTTTTG	*Wnt4*	F: AGACGTGCGAGAAACTCAAAG
	R: TTGGCTGAGTGGTAGAGTCCC		R: GGAACTGGTATTGGCACTCCT
Ccl2	F: TGCCCTAAGGTCTTCAGCAC	*Wnt5a*	F: CAACTGGCAGGACTTTCTCAA
	R: AAGGCATCACAGTCCGAGTC		R: CATCTCCGATGCCGGAACT
Ccl7	F: GCTGCTTTCAGCATCCAAGTG	*Tnfsf11*	F: CAGCATCGCTCTGTTCCTGTA
	R: CCAGGGACACCGACTACTG		R: CTGCGTTTTCATGGAGTCTCA

Supplementary Table 2. Antibodies for immunochemistry

Antibodies	Source	Identifier	Dilution
Krt14	Wuhan Dia-an Biotechnology Co., Ltd.	Cat#C0740	1:200
Krt8	DSHB	Cat#TROMA-I	1:1000
Ki67	BioLegend	Cat#151202	1:400
α- tubulin	Proteintech	Cat#11224-1-AP	1:200
β-casein	Santa Cruz Biotechnology	Cat#sc-166530	1:50
